# IS THERE NEURAL AND FUNCTIONAL RECOVERY AFTER CLIP REMOVAL IN CERVICAL EXPERIMENTAL SYMPATHECTOMY?

**DOI:** 10.1590/0102-672020210002e1582

**Published:** 2021-10-18

**Authors:** Carlos Hespanha MARINHO-JUNIOR, Nicolau Gregori CZECZKO, Victoria Langer CECHIN, Joao Otavio Varaschin ZENI, Jurandir Marcondes RIBAS-FILHO

**Affiliations:** 1 Mackenzie Evangelical Faculty of Paraná, Curitiba, PR, Brazil; 2 University Evangelical Mackenzie Hospital, Curitiba, PR, Brazil; 3 Positivo University, Curitiba, PR, Brazil

**Keywords:** Sympathetic nerve, Clipping, Nerve damage, Sympathetic block, Polymer clip, Sympathectomy reversal, Rabbits, Nervo simpático, Clipagem, Lesão nervosa, Bloqueio simpático, Cipe de polímero, Reversão de simpatectomia, Coelhos

## Abstract

**Background:**

The surgical treatment of hyperhidrosis by thoracic sympathectomy has brought, in addition to symptomatic relief for many, its main adverse effect: compensatory or reflex sweating. The clipping technique in place of the sympathetic nerve section gave rise to the hope of reversibility, but the positive results showed to be quite divergent, evidencing the academic deficiency regarding the study of this phenomenon.

**Aim:**

To observe micro and macroscopic damage caused by the polymer clip on sympathetic nerve of rabbits seven days after their clipping and the findings after three weeks of clip removal.

**Method:**

In this experimental study, 20 rabbits were divided into two groups of 10, group 1 (clipping) and group 2 (de-clipping). The right cervical sympathetic nerve of all animals was clamped with polymeric clip, and in group 2 the nerve was unclipped seven days later. Group 1 rabbits were induced to death on the 7th postoperative day, and group 2 on the 21st after removal of the polymer clip. Macroscopic variables were: clip appearance, presence of discontinuity lesion, infection and adhesions around the nerve. H&E was used in the evaluation of the phases and degree of the inflammatory process and presence of necrosis, and picrosirius red F3BA for quantification of collagen.

**Results:**

The cervical sympathetic nerve was intact, without necrosis or infection in all animals of the experiment; there were adhesions in both groups, being minimal in eight animals of each group and moderate or intense in two; the clip was completely closed in all animals at the 7th postoperative day; the inflammatory process shown was chronic, with monomorphonuclear predominance. There was no significant difference between groups regarding the intensity the inflammatory process, but the amount of collagen type I and type III was significantly higher in group 2.

**Conclusions:**

The injury caused by the polymer clip on the sympathetic nerve may be reversible, allowing functional return in the areas involved in the simulated cervical sympathectomy. Clipping of the cervical sympathetic nerve using a polymer clip does not cause discontinuity injury.

## RESUMO

***Racional*:**

O tratamento cirúrgico da hiperidrose pela simpatectomia torácica trouxe além do alívio sintomático para muitos, também seu principal efeito adverso: o suor compensatório ou reflexo. A técnica de clipagem do nervo simpático no lugar de sua secção deu margem à esperança de reversão do procedimento; porém, os resultados mostram-se bastante divergentes e pouco conclusivos

**Objetivo:**

Observar a lesão provocada pelo clipe de polímero em nervo simpático de coelhos sete dias após sua clipagem, comparando-a com os achados após três semanas da retirada do clipe.

**Método:**

Estudo experimental, com amostra formada por 20 coelhos, divididos em dois grupos de 10, sendo o grupo 1 chamado clipagem e o 2, desclipagem. Todos foram submetidos à clipagem do nervo simpático cervical direito com clipe polimérico, e no grupo 2 realizou-se a desclipagem sete dias após. Os coelhos do grupo 1 foram submetidos à eutanásia no 7º dia de pós-operatório, e os do grupo 2 no 21º dia após a remoção do clipe. Observou-se na macroscopia o aspecto do clipe, a presença de lesão de descontinuidade, infecção e aderências ao redor do nervo. Para estudo microscópico utilizou-se a coloração H&E na avaliação das fases, grau do processo inflamatório e presença de necrose, e a de picrosirius red F3BA para quantificação de colágeno.

**Resultados:**

O nervo simpático cervical foi identificado íntegro, sem necrose ou infecção em todos os animais do experimento; havia aderências em ambos os grupos, sendo mínimas em oito de cada grupo e moderadas ou intensas em dois; em toda a amostra o clipe encontrava-se completamente fechado no 7º dia de pós-operatório; o processo inflamatório presente foi do tipo crônico, com predomínio monomorfonuclear, não se observando diferença significativa em relação ao grau do processo inflamatório entre os grupos; porém, a quantidade de colágeno tanto do tipo I quanto do tipo III foi significativamente maior no grupo 2.

**Conclusões:**

A lesão provocada pelo clipe de polímero em nervo simpático pode ser reversível possibilitando o retorno funcional nas áreas envolvidas na simpatectomia cervical simulada. A clipagem do nervo simpático cervical com uso de clipe de polímero não causa lesão de descontinuidade.

## INTRODUCTION

Galeno, in the 2^nd^ century, was the author of the first description, although erroneous, of the anatomy of the sympathetic trunk. The proper anatomical description of this nerve was not defined until the end of the 18^th^ century^14^.

Considering that it was the physiological tests performed by Gaskell, at the beginning of the 20^th^ century, that enabled the anatomophysiological mapping of the autonomic nervous system, it is concluded that the sympathectomies performed before that period had their scientific obscure indications, as to treat goiter thyroid, epilepsy and glaucoma^10^. Only in 1920, Kotzareff^5^ performed the first sympathectomy to treat hyperhidrosis of the upper limbs. At that time, however, considered an indication of little importance for a surgery, with the advent of video-thoracoscopy in the 1990’s^9^, the sympathectomy became widely used, with its most frequent side effect being compensatory or reflex sweat, whose incidence varies from 47-99%^5^ ^,^ ^6^ ^,^ ^8^ ^,^ ^13^ ^,^ ^18^ ^,^ ^21^.

Based on experimental studies that demonstrate that a permanent compression on a peripheral nerve with a force greater than 44 grams-force blocks the conduction of the nervous impulse, added to the possibility of reversing the clipping to reverse the intolerable compensatory sweat, several groups started to use the block sympathetic nervous system through clipping for the treatment of primary hyperhidrosis. However, the success rates of reversing the compensatory sweat with the removal of the clip show very divergent rates, ranging from 15-100%^16^.

Seeking light in science, experimental studies have been carried out; however, they are very scarce and with quite divergent results. This lack of experiments, as well as the absence of convincing and robust responses in the literature on the reversal of the injury caused by clipping after removal of surgical clips from the sympathetic nervous system in both humans and experimental animals, motivated this research to be done. 

## METHOD

This research was carried out at the Institute of Medical Research (IPEM) home of Post-Graduation Program in Principles of Surgery, Evangelical Mackenzie Faculty of Paraná, Curitiba PR, Brazil, and at the vivarium of Positivo University, Curitiba, PR, Brazil. The research was only carried out after being submitted to the approval of the Committee for Ethics in Animal Research of the Evangelical Mackenzie Faculty of Paraná in compliance with the ethical principles recommended by the Brazilian Society of Laboratory Animal Science (SBCAL) and Federal Law No. 11,794 (08/10/2008).

### Sample

Twenty male rabbits (Oryctolagus cuniculus) were used, with body weight between 1850-2000 g. They received commercial rations (Nuvital®, Quimtia Brazil S.A) and were kept in air-conditioned rooms with a temperature of 18-22°C, 65% relative air humidity and a 12 h photo dark period.

The animals were sent to the experiment room 15 days before the start of operations. After the acclimatization period, they were weighed, identified and kept in individual cages. Identification was performed according to the standard adopted in the vivarium, using numbering in the ears.

### Division in groups

The 20 rabbits were divided into two groups: group 1 or clipping group, where the right cervical sympathetic nerve was clipped with a 5 mm polymer clip (Hem-o-lok^®^, Teleflex Medical), being the animals numbered 1 at 10, and group 2, or decliping group, numbered from 11 to 20, where the same clipping of the right cervical sympathetic nerve of the animals of group 1 was performed, but on the 7^th^ postoperative day they underwent a second procedure, in which the clip was removed. A single clip per animal was used in both groups.

### Anesthesia and surgical procedure

As a pre-anesthetic drug, 1 mg/kg midazolam was given intramuscularly. After reached the sedative plane, the animals received a combination of 5 mg/kg of 2% xylazine hydrochloride with 35 mg/kg of 10% ketamine hydrochloride intramuscularly. The venous access was made to the superficial vein of the ear after obtaining the anesthetic plan. For puncture of venous access was used a 22G polyurethane peripheral venous cateter. The rabbit was positioned on the surgical table where it received inhaled isofluorane through facial mask to maintain the anesthetic plane. The oxygen saturation and the heart rate were monitored during all the perioperative period in a pulse oximeter.

Once in dorsal decubitus, with a slight cervical extension, hair removal of the cervical region was performed by pulling them after application of shaving cream. Antisepsis of the skin was fulfilled with solution of 1% iodine polyvinylpyrrolidone-iodine.

**FIGURE 1:**
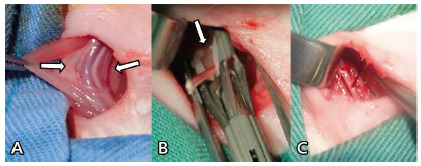
A) Cervical neurovascular bundle (between the arrows); B) nerve clip (arrow); C) demarcation of the clipping site

The surgical technique consisted of: 1) a median longitudinal cervical incision of about 1.5 cm; b) blunt dissection between the superficial and middle lamina of the cervical fascia to identify the infrahyoid muscles; 3) opening of the pre-tracheal muscles longitudinally by divulsion in its midline and identification of the trachea; 4) identification of the right cervical vasculo-nervous bundle in right paratracheal topography (Figure 1A); 5) individualization of the right sympathetic nerve; 6) clipping between the 1/3 caudal and 2/3 cranial in both groups (Figure 1B); 7) closure of the surgical incision with single stitches of monofilament nylon.

The animals of group 2 or declipping were submitted to cervicotomy on the 7^th^ postoperative day following the same technique used in the first procedure, identifying the polymer clip releasing it from the nerve using scissors. The location of the clipping was demarcated (Figure 1C).

At the end of the surgical procedure each rabbit received 0.8 mg/kg of butorphanol tartrate subcutaneously every 8 h for two days, and a single dose of 0.5 ml broad-spectrum veterinary pentabiotic. During the postoperative period, the amount of water and food ingested by the rabbits was observed to determine the level of comfort.

### Euthanasia

The rabbits in group 1 were dead at seven days postoperatively by rapid intravenous injection of 10 mg/kg of sodium thiopental 2.5%; already, the group 2 was sacrificed on the 28^th^ day after the first operation, that is, in 21^st^ post-desclipagem day.

### Sample collection

Once the time of the experiment was completed, the animals were submitted to euthanasia, and the right cervical sympathetic chains were dissected using surgical material. Afterwards, its cranial and caudal end was marked with 3/0 cotton yarn and left at the caudal end to the area to be studied microscopically, a shorter strand of yarn and a longer stump in the cranial. The pieces were placed in flasks containing 10% formalin solution, identified with the animal number and date. They were then referred to the laboratory of animal pathological anatomy.

### Macroscopic evaluation

For the evaluation of the sympathetic nerves, the clinical analysis was performed in the natural, and photographed.

The criteria for objective evaluation of the process triggered by the lesions on the 7^th^ postoperative day were: identification of the cervical sympathetic nerve (grade 0=absent, grade 1=present); macroscopic nerve discontinuity (grade 0=absent, grade 1=present); aspect of the clip on the nerve (grade 0=hermetically sealed, grade 1=partially open, grade 2=fully open) and the presence of infection around the nerve (grade 0=absent, grade 1=mild infection, grade 2=abscess). For adhesion evaluation, was adopted the methodology already known^15^: grade 1=minimum adhesions; grade 2=moderate adhesions; grade 3=intense adhesions.

### Microscopic evaluation

Optical microscopy processing of the collected material was performed at the nerve edges of the site where the clipping was performed (group 1) and at the site of the declipping (group 2).

Once fixed, the specimens were dehydrated with successive passages in ethyl alcohol solutions in increasing concentrations of 70%, 80%, 90% and absolute. Afterwards, they were diaphanized in xylol, paraffin embedded and blocked. Microtomes were cut 5 μm thick and later mounted on 75x25 mm slides, which were duly identified with the animal number and stained by H&E and picrosirius red F3BA (PSR).

#### H&E staining

The stained histological sections were analyzed by binocular optical microscope, with flat, apochromatic lenses, by a pathologist who did not know the group to which the studied slide belonged. This method had the objective of evaluating the type and quantity of the predominant cells in the inflammatory reaction (polymorphonuclear and monomorphonuclear infiltrates) and presence of necrosis. The presence of edema, congestion, hemorrhage and neutrophilic cells were indicative of an acute inflammatory process, as a chronic inflammatory mononuclear infiltrate. Necrosis characterized absence of regeneration, so, the end result of a degenerative process.

The data obtained by the H&E technique were classified based on the histopathological characteristics, according to the intensity in which they were found (Table 1) in case of an acute inflammatory process and the same with the chronic process (Table 2). By assigning index to the histological findings, they were converted into quantitative variables.

**TABLE 1:** Classification of the acute inflammatory process based on histopathological characteristics

Histopathological features	Intensity of findings			
	Abundant	Moderate	Mild	Absent
Neutrophils	3	2	1	0
Edema	3	2	1	0
Congestion	3	2	1	0

**TABLE 2:** Rating chronic inflammatory process based on histopathological features

Histopathological features	Intensity classification
Mild Wallerian degeneration Mild monomorphonuclear inflammatory infiltrate Minimal axonal swelling	Discreet/minimum=1
Moderate Wallerian degeneration Moderate monomorphonuclear inflammatory infiltrate Medium axonal swelling	Moderate/medium=2
Marked Wallerian degeneration Marked monomorphonuclear inflammatory infiltrate Maximum axonal swelling	Marked/maximum=3
Nerve necrosis	Nerve necrosis=4

#### Picrosirius Red F3BA (PSR) staining

The histological sections aimed at the identification and quantification of collagen, mature and immature, by polarized light microscopy technique and computerized morphometric analysis.

The slides were read using the Pro-image-plus 4.5^®^ program for Windows coupled to the optical microscope, that was previously regulated in square micrometer with a 40-fold objective. With capture camera, images were sent to a color monitor, frozen and scanned by Oculus TCX^®^ digital imaging board. Three collagen measurements were performed in each longitudinal field and three in each cross field. The measurements were transferred to the Excel Windows program.

After the observation of the field in the area of the blade stained by the PSR, the polarization was performed. All non-collagenous substance was stained black. Mature or type I collagen was stained in yellow, red-orange and red, while type III or immature collagen was stained green. These colors were selected through the program for quantification and summation of the selected area in square micrometer. The six measures performed were summed up after a simple mean was calculated. The data were then transported to the Windows Excel program and placed in a table for statistical analysis.

With the same Pro-Image Plus application, the total area (in square micrometer), percentages of type I and type III collagen were analyzed. The thicker and more strongly birefringent collagen fibers dyed with orange and red (type I), and the finer and less birefringent fibers with green (type III). An average of these percentages was obtained in each histological section.

All slides were evaluated under the same setting conditions, within the parameters required by said application.

### Statistical analysis

The statistical treatment was adequate according to the nature of the data analyzed and the size of the clipping and declipping groups. The t-test was used in the comparisons of types I and III collagens between groups. Fisher’s test was used to compare the classification of perineural adhesions and the type of chronic inflammatory process between groups. The analysis was descriptive for presence of inflammatory process and necrosis due to the frequencies presented in the groups. The index for statistical significance was set at 0.05 or 5%.

## RESULTS

### Macroscopic evaluation

**FIGURE 2:**
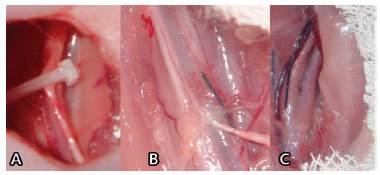
A) Macroscopic nerve integrity at operative time for clip removal and the appearance of the closed clip; B) adhesions classified as minimum; C) adhesions classified as moderate or severe

**FIGURE 3:**
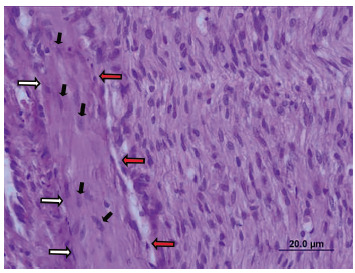
Chronic inflammatory process to the H&E staining in longitudinal cut of the sympathetic nerve: the white and red arrows delimit the perineurium, in which the proliferation of the Schwann cells (black arrows) is observed; to the right of the red arrows is the endoneurium, with a marked mononuclear inflammatory infiltrate (100x)

In reoperations and necropsies the cervical sympathetic nerve was easily identified. In all animals of both groups: 1) no lesion of nerve discontinuity was observed; 2) the clip was completely closed on the 7^th^ postoperative day (Figure 2A); 3) no cases of infection or abscess were observed around the nerve.

About the presence of adhesions around the right cervical sympathetic nerve, in both groups - clipping and declipping - adhesions were formed, being classified as minimal (Figure 2B) in eight animals of each group and moderate or intense (Figure 2C) in two of each group.

### Microscopic evaluation

#### Presence of necrosis and evaluation of the inflammatory process

The presence of a chronic inflammatory process was observed in all animals, with a predominance of monomorphonuclear infiltrate (Figure 3) and in none was the presence of necrosis.

Regarding the degree of the inflammatory process, one animal in the clipping group was classified as moderate and in the other nine as a discrete. In all of unlatch group the inflammatory process was classified as discrete. There was no significant difference in the degree of inflammatory process between the groups (p=0.5).

#### Collagen evaluation

Tables 3 and 4 demonstrate the means in square micrometer of type I and III collagen, respectively, and comparing them between the groups.

**FIGURE 4:**
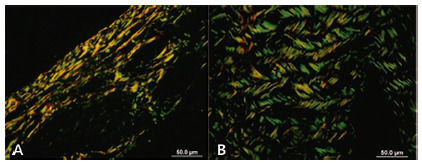
Photomicrography of the cervical sympathetic nerve in PSR staining with polarized light: A) longitudinal section (20x); B) cross section (20x)

A significant difference was observed between the areas of type I collagen, in square micrometer, between groups (p<0.0001), as well as between areas of type III collagen (p<0.0001). Figure 4 shows collagen presentation in PSR staining.

**TABLE 3:** Statistical analysis of the means of collagen type I between the clipping and declipping groups (t-test)

Groups	n	Collagen type I				p
		min - max	mean	±	sd	
Clipped	10	3,16 - 24,26	10,83	±	6,85	< 0,0001
Unclipped	10	15,78 - 42,79	32,29	±	7,95	

**TABLE 4:** Statistical analysis of the means of collagen type III between the clipping and de-clipping groups (t-test)

Groups	n	Collagen type III				p
		min - max	mean	±	sd	
Clipped	10	14,50 - 39,76	24,79	±	7,69	< 0,0001
Unclipped	10	30,67 - 51,59	45,12	±	6,4	

## DISCUSSION

### Experimental animal

Studies about the anatomy of the cervical sympathetic nerve in experimental animals are rare in the literature. Kalsey *et al.* ^11^ in a research where they compared the anatomy of the cervical sympathetic nerve of dogs, albino rats, lizards, rabbits, chickens and guinea pigs, observed that this nerve usually crosses the lower half of the neck in isolation from the vagus nerve, except in the dog. They also found that in rats, rabbits and guinea pigs, the cervical sympathetic nerve had no subdivisions. Due to the ease of obtaining, as well as standardization of age and weight, housing manner and availability of feed, caliber of its nerve and allowing its easy identification, the rabbit was chosen in the present experiment.

During the experiment, rabbits were shown to be docile and easy to handle, and although they were cited as very sensitive to surgical^4^ and anesthetic procedures, there were no preoperative, trans and postoperative complications and no animals died due to anesthetic or surgical complications.

### Inflammatory process and healing after sympathetic nerve injury

Classic works^1^ ^,^ ^7^ have already demonstrated that the inflammatory activity after nervous injury is intense, associated with the process of wallerian degeneration.

Quantitative studies of sympathetic nerve scarring indicators are rare in the literature. A research in Brazil^17^ developped an experimental model in pigs to identify the anatomical regeneration of communicating branches of the thoracic sympathetic nerve after its section - ramicotomy. As a conclusion, they reported that up to the 15^th^ postoperative day the inflammatory reaction is intense and no anatomical neural regeneration was observed; however, on the 45^th^ postoperative day anatomical neural regeneration was established.

Marinho Jr. *et al.* ^14^ comparing inflammatory process after sectioning and clipping using titanium clip on the sympathetic nerve, also observed intense inflammatory reaction in the animals on the 7^th^ postoperative day.

Arantes-Marinho^2^, for the first time described experimental use of Hem-o-lok^®^ for sympathetic nerve block, comparing it with the titanium clip. They observed predominance of an intense inflammatory process with the titanium; however, in all animals where the Hem-o-lok^®^ was used the inflammatory process was discrete or moderate, which was also found here.

Observational studies on the effect of clipping of the thoracic sympathetic chain^8^ ^,^ ^13^ ^,^ ^18^ confirm its effect on nerve conduction blockade, however without histological demonstration of the lesion provoked.

Marinho Jr. *et al.* ^14^ did not identify significant differences in the inflammatory process caused by the clipping of the sympathetic nerve compared to the one with its section. However, due to the absence of continuity lesion observed by the clipping lesion, they suggested that studies on the inflammatory process at different periods after the removal of the compressive effect of the clipping could demonstrate a possible involution of the inflammatory process, associated or not to anatomofunctional regeneration.

Arantes-Marinho^2^ found that the formation of adhesions around the cervical sympathetic nerve was significantly lower when using Hem-o-lok^®^ compared to the conventional titanium clip, which suggests greater technical ease in cases where a new intervention is necessary. The present study also refers to the predominance of minimal perineural adhesions.

### Collagen evaluation

Oliveira *et al.* ^17^ observed that there was an increase in both type I and III collagen fiber levels, which was constant up to the 90^th^ postoperative day. From then on, they observed a progressive increase in the amount of collagen fibers, being their greatest peak of neural area occupancy on the 135^th^ postoperative day.

Marinho Jr. *et al.* ^14^ and Arantes-Marinho^2^ also demonstrated a deposit of collagen in area of sympathetic nerve damage, with predominance of type I collagen in relation to type III, even in the early stage.

This research, therefore, corroborates with these studies demonstrating that there is collagen deposition in area of the lesion provoked in the sympathetic nerve, being already in the early stage, the presence of type I collagen is greater than that of type III.

The lower deposit of type III or immature collagen in relation to type I or mature, short period of the nerve injury has already been reported confirming that in the initial phase of wallerian degeneration^20^, after peripheral nerve injury, absorption of part of the endoneurium - constituted by reticular fibers - the main composition of which is type III collagen.

### Perspectives

There are few reports in the literature as well as the clinical series evaluating clip removal after sympathetic block for the treatment of hyperhidrosis. It is not possible to explain why the clinical results are so discrepant. Sugimura *et al.* ^21^ reported the largest series of cases, with longer follow-up, which was greater than five years. They observed reduction of compensatory sweat in approximately half of the patients; however, found no explanation for the fact that in 40% of the patients who experienced compensatory sweat reduction there was no recurrence of the original hyperhidrosis. Because there is no reproducible animal model to evaluate the mechanism of hyperhidrosis and to validate the concept of sympathetic blockade and its reversibility, they have suggested the need for experimental studies to do so.

The present study developed an experimental model with the potential to validate the concept of reversibility of sympathetic blockade. It is suggested to carry out research comparing the inflammatory process and the quantification of collagens in different periods after the removal of the compressive effect of the clip, aiming to demonstrate the possibility of partial or total anatomical healing or regeneration of the nerve, as well as its association or not to a greater or lesser deposit of collagen fibers. It also makes possible the evaluation of the existence or not of relation between the types of collagen in the process of healing of the sympathetic nerve.

It also demonstrates the need to evaluate new materials^A^ and technologies, since the use of materials that are more inert to the organism can lead to less adhesion formation, facilitating re-approaches and, therefore, generating less surgical trauma.

## CONCLUSIONS

The injury caused by the polymer clip on the sympathetic nerve can be reversible allowing the functional return in the areas involved in the simulated cervical sympathectomy. Clipping of the cervical sympathetic nerve using a polymer clip does not cause discontinuity injury.
